# A reminder—peptidoglycan cell walls indeed occur in the archaeal domain, specifically in the members of Methanobacteria and Methanopyri classes

**DOI:** 10.3389/fmicb.2024.1329047

**Published:** 2024-05-09

**Authors:** Biswarup Mukhopadhyay

**Affiliations:** Department of Biochemistry, Virginia Tech, Blacksburg, VA, United States

**Keywords:** archaea, methanogen, cell wall, peptidoglycan, misconception, reminder

In spite of a clear molecular explanation presented more than four and half decades ago (Kandler and König, 1978), a misconception that archaea lack peptidoglycan and some of these organisms carry pseudomurein or pseudopeptidoglycan has proliferated both in peer-reviewed publications and textbooks and online educational materials (Albers and Meyer, 2011; Visweswaran et al., 2011; Rodrigues-Oliveira et al., 2017; Bruslind, 2019; Baker et al., 2020; Madigan et al., 2021; Medvedeva et al., 2023; Salas et al., 2024) (bio.libretexts.org). This article is intended to describe how this misconception arose and when and with what rationale a clear attempt was made to remove it.

In 1977, based on a 16S rRNA-based phylogenetic analysis of several methanogens, Woese and Fox identified archaebacteria as a prokaryotic group that is distinct from bacteria (Woese and Fox, 1977); later, archaebacteria were renamed as archaea (Woese et al., 1990). The specialized cell walls of methanogens that were assumed to lack peptidoglycan (Kandler and Hippe, 1977) were a key feature supporting the declaration (Fox et al., 1977). In 1978, Kandler and König reported that the rigid components of the cell walls of six species of *Methanobacterium*, which are currently known as *Methanobacterium formicicum, Methanothermobacter thermautotrophicus, Methanobrevibacter ruminantium, Methanobrevibacter arboriphilus*, and *Methanobacterium bryantii*, and members of the order Methanobacteriales within the class of Methanobacteria (Rinke et al., 2021), lacked muramic acid or D-amino acids, which are typical constituents of bacterial peptidoglycan. Instead, they consisted of L-amino acids (Lys, Glu, and Ala) and N-acetylglucosamine or N-acetylgalactosamine (Kandler and König, 1978). For apparent similarities to peptidoglycan, which is also called “murein” for one of its key components, muramic acid, Kandler proposed that this structure of *Methanobacterium* should be termed “pseudomurein” (Kandler, 1979). König and Kandler showed that, in *Methanobacterium thermoautotrophicum* (currently, *Methanothermobacter thermautotrophicus*), the peptide moiety of this polymer contains an unusually high number of ε- and γ-bonds, and it is coupled to the glycan strand via a glutamyl residue (Konig and Kandler, 1979), and N-acetyltalosaminuronic acid is a component of the glycan strand (König and Kandler, 1979; König et al., 1983). These observations solidified their proposal that pseudomurein is distinct from peptidoglycan and is a result of convergent “invention” (König and Kandler, 1979). They showed that the glycan strands of pseudomurein are composed of alternating N-acetylglucosamine or N-acetylgalactosamine and N-acetyltalosaminuronic acid residues linked through β-1,3 glycosidic bonds, whereas, in the strands of bacterial peptidoglycan, one finds β-1,4-linked N-acetylglucosamine and N-acetylmuramic acid units (König et al., 1982) (Figure 1).

**Figure 1:**
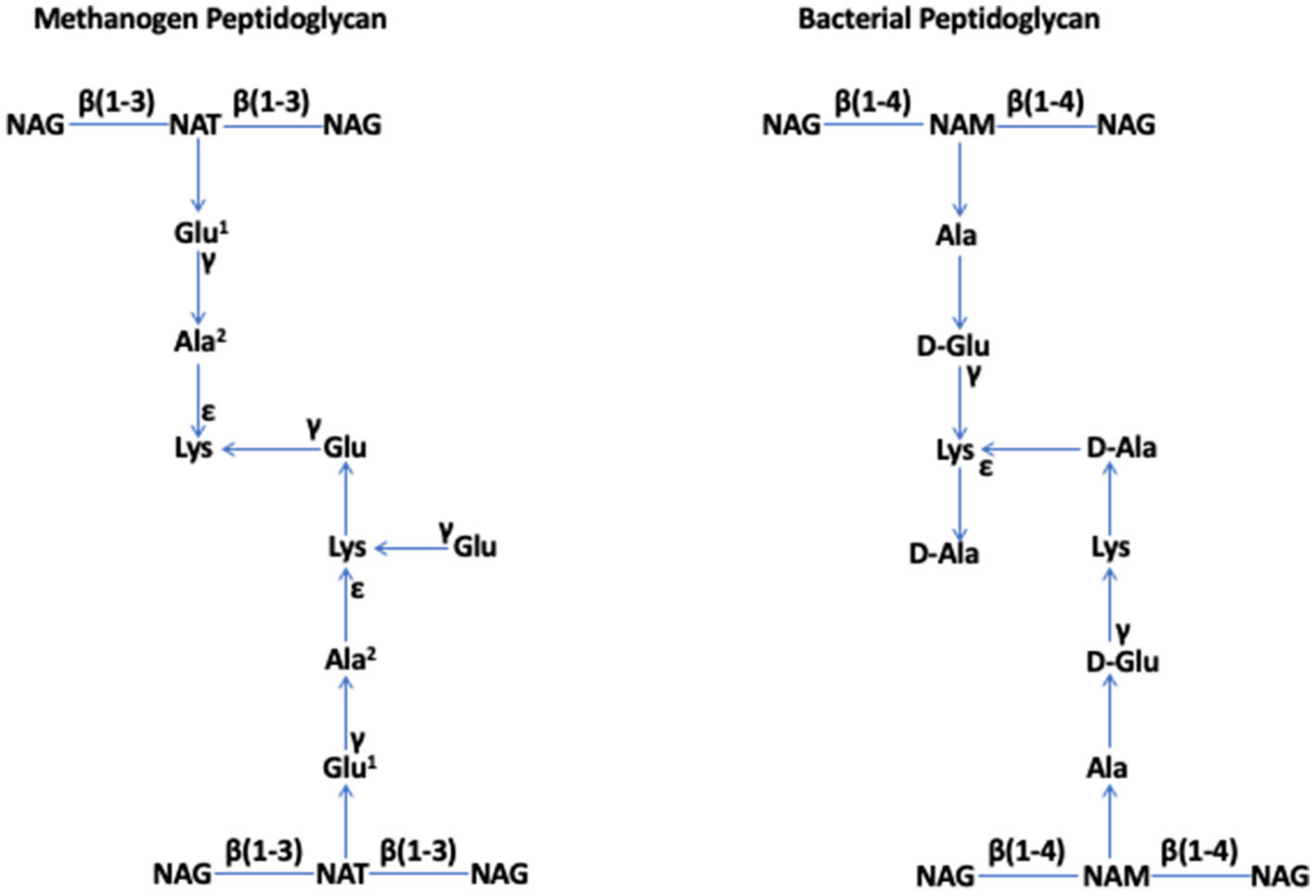
Methanogen and bacterial peptidoglycan (Kandler, 1993; Kandler and Konig, 1993; Albers and Meyer, 2011; Kandler and König, 2014; Tashiro et al., 2017; Subedi et al., 2021). NAG, N-acetylglucosamine; NAT, N-acetyltalosaminuronic acid; NAM, N-acetylmuramic acid. Special features. *Methanogen peptidoglycan*: β (1–3) bonds in the glycan strand and L-amino acids in the peptide moiety, where Glu^1^ and Ala^2^ can be replaced by Asp and Thr or Ser, respectively, in some members. *Bacterial peptidoglycan*: β (1–4) bonds in the glycan strand and both L- and D-amino acids in the peptide moiety. The figure has been adapted and modified from Subedi et al. (2021).

The aforementioned effort to define the rigid component of the methanobacterial cell wall as a novel entity *came to face reality* when conformational energy calculations suggested that murein and pseudomurein share a similar three-dimensional (3D) architecture (Leps et al., 1984a,b). Kandler and König recognized that their concept that the cell walls of methanogens lacked peptidoglycan (Kandler and König, 1978) was not correct and they issued a correction: “*Such a polymer must also be considered as a peptidoglycan, a very general chemical term”* (Kandler and König, 1985); a similar clarification has appeared more recently as well (Claus and König, 2010). A peptidoglycan cell wall has also been found in *Methanopyrus kandleri*, the only characterized representative of *Methanopyrus* (of the Methanopyri class and Methanopyrales order) (Kurr et al., 1991).

The above-described findings need to be considered in the context that, even within the bacterial domain, the peptidoglycan structure varies (Vollmer, 2008). *Thus, the versions of this polymer that one finds in the methanogens of the orders of Methanobacteriales and Methanopyrales are not pseudomurein as it does not contain muramic acid but simply another type of peptidoglycan, where glycan strands are connected by peptide linkages. The name archaeal peptidoglycan that has been used recently (Khairunisa et al.*, *2023**) is appropriate in this context*.

To summarize, in most methanogens and almost all other archaea, a crystalline surface layer (S-layer) composed of one or two glycoproteins encases the cytoplasmic membrane formed by either a diether lipid bilayer or a tetraether lipid monolayer (Albers and Meyer, 2011; Klingl et al., 2019; Meyer et al., 2022; van Wolferen et al., 2022). In some cases, a protein sheath and a layer of methanochondroitin (a proteoglycan made of N-acetylgalactosamine and glucuronic acid) are found to occur over the S-layer (Albers and Meyer, 2011; Klingl et al., 2019; Meyer et al., 2022). In contrast, in a limited number of methanogens that belong to the genera of *Methanobacterium, Methanothermobacter, Methanosphaera*, and *Methanobrevibacter* (of the class of Methanobacteria and the order of Methanobacteriales) and *Methanopyrus* (of the Methanopyri class and the Methanopyrales order) of the Methanobacteriota phylum (Rinke et al., 2021), a rigid sacculus made of glycan strands connected by peptide linkages or peptidoglycan takes the place of the S-layer as described above (Albers and Meyer, 2011; Klingl et al., 2019; Meyer et al., 2022; van Wolferen et al., 2022); in *Methanothermus*, which also belongs to the Methanobacteriales order, an S-layer is placed over the peptidoglycan.

The recognition that certain archaea contain peptidoglycan brings the studies on the cell walls of *Methanobacteria* and *Methanopyri* to a larger arena, and consequently, this recognition will accelerate the discovery process that will provide products of applied value, such as improved methods for environmental detection, creation of archaeal-specific antibiotics, and improvements in genetics and cell biology research. These advances would facilitate the development of better methods for mitigating methane emission from livestock, manipulating human gut metabolism/microbiome toward better health, and improving processes for methane production and biofuel from renewable resources. *Methanobacteriales* constitute a great portion of the bovine rumen and human gut methanogen population (Eckburg et al., 2005; Borrel et al., 2020; Khairunisa et al., 2023) and are attractive for industrial bioproduction of methane, as well as amino acids (Pappenreiter et al., 2019; Pfeifer et al., 2021; Taubner et al., 2023).

## Author contributions

BM: Writing – original draft, Conceptualization.
